# Gender differences in the effect of a 0.11% breath alcohol concentration on forward and backward gait

**DOI:** 10.1038/s41598-022-23621-y

**Published:** 2022-11-05

**Authors:** Marta Gimunová, Michal Bozděch, Jan Novák, Tomáš Vojtíšek

**Affiliations:** 1 Department of Kinesiology, Faculty of Sport Studies, Masaryk University, Brno, Czech Republic; 2 Department of Gymnastics and Combatives, Faculty of Sport Studies, Masaryk University, Brno, Czech Republic; 3 Department of Forensic Medicine, An Shared Institution of St. Anne’s Faculty Hospital and the Faculty of Medicine, Brno, Czech Republic

**Keywords:** Physiology, Health occupations

## Abstract

Alcohol contributes to a large number of diseases and health conditions related to injuries. The aim of our study was to evaluate gender differences in forward and backward gait when sober and at a breath alcohol concentration (BrAC) of 0.11%. Fifty females and fifty males participated in our study. The gait analysis was performed twice, when sober and after drinking a given amount of vodka mixed with orange juice. Under both conditions, participants were asked to walk forward and then backward on a Zebris platform. Multivariate analysis and the Mann–Whitney U test were used to compare the differences between genders when walking forward and backward. The Wilcoxon Signed Ranks test was used to compare the differences between 0.00% BrAC and 0.11% BrAC. Spearman’s Rho was used to analyze the relationship between the AUDIT score, anthropometrical characteristics and the subjective score of drunkenness and gait parameters. The results show different strategies to improve stability during gait in women and men when intoxicated with alcohol. When intoxicated, males in forward gait increase their stability by increasing their foot rotation, while females increase their step width. A decrease in balance-related variables was observed in females when walking backward with a BrAC of 0.11%. Additionally, females tended to perform an increase in balance-related gait variables when subjectively feeling more drunk in both forward and backward gait. Different strategies to maintain stability during gait were observed in women and men. The results of our study show that alcohol intoxication has a greater impact on gait in females who tended to perform an increase in balance-related variables with an increase in their subjective score of drunkenness.

## Introduction

Alcohol contributes to more than 200 diseases and injury-related health conditions^1^. Approximately 3 million global deaths and 5.1% of disability-adjusted life years (132.6 million, the sum of years of life lost to premature mortality) were attributed to alcohol consumption in 2016^2^.

Gender differences in several parameters of alcohol metabolism have been described in previous studies. Women differ from men in having lower alcohol dehydrogenase activity in the stomach which increases the bioavailability of ethanol. A decrease in the volume of ethanol distribution that is 7.3% smaller compared to men results in a higher blood alcohol level in women. Women show a 10% higher rate of ethanol oxidation and elimination in the liver compared to men, and their gastric emptying rate of alcohol is 42% slower compared to men. These differences lead to an equal alcohol intake resulting in a higher blood alcohol concentration in women^3,4^. Furthermore, gender differences have also been observed in the frequency and quantity of alcohol intake. A cross-cultural study shows that men exhibit higher alcohol drinking frequencies and quantities^5^. However, in the last decade the rates of alcohol use, high-risk alcohol drinking and alcohol use disorder have increased significantly in women (by 16%, 58% and 84%, respectively) compared to a smaller increase observed in men (by 7%, 16% and 35%, respectively)^6^. Gender differences in various neurotransmitter systems may play a critical role in the risk of developing alcohol use disorders^7^.

Forward gait is one of the most basic movements in daily life. The hypothalamus and the midbrain control the onset of gait, while the cerebellum and brainstem take control during gait^8^. The cerebellum is particularly vulnerable to alcohol intoxication and the toxic effects of ethanol may result in ataxic gait^9^. Cerebellar gait ataxia, walking with “tipsy” steps, is characterized by a reduced cadence and increased balance-related variables such as step width and foot rotation angle^10^. Furthermore, the forward gait after moderate alcohol intoxication appears similar to the gait of the elderly with a higher risk of falls. It demonstrates a decrease in stride length and gait velocity^8^. Changes in forward gait parameters have been observed from 0.4 mg alcohol/L^11^.

Backward gait is a daily activity, when opening a door for example, and the reduced ability to take steps backwards leads to an increased risk of falling^12^. Forward and backward gait have the same basic neural mechanism; however, backward gait poses a greater challenge to the nervous system^13^. During backward gait, people need to rely more on other senses (i.e. auditory or sensory systems) rather than the visual system^14,15^. The central nervous system (CNS) processes sensory information, and when one or more sensors are unavailable or disrupted (e.g. vision in backward gait) the CNS can partly compensate by sensory reweighting^16,17^. Acute alcohol intoxication is characterized by increased visual dependency compared to vestibular information^16^ which may lead to greater gait impairment during backward gait compared to forward walking when intoxicated with alcohol. However, the authors do not know of any previous study on the effect of alcohol intoxication on backward gait.

Gender differences in gait related to anatomical differences between genders (e.g. a wider pelvis and its effect on hip motion in females) have previously been described. Forward gait in women is characterized by shorter stride length, higher cadence and narrower step width as compared to men^18–20^. When walking backward, a slower gait velocity and a shorter stride length were observed in women as compared to men^19^.

Previous studies focusing on gait impairments caused by alcohol intoxication have included less than 25 participants and have not focused on gender differences^8,11,21^, despite previous research showing gender differences in behavior outcomes resulting from alcohol intoxication (e.g. McCabe^22^). In our study, we focus on equal gender representation of participants as there remains significant underrepresentation of women in sport and health-related studies^23^. The aim of our study was to analyze the forward and backward gait when sober and at a 0.11% breath alcohol concentration (BrAC) and to assess the gender differences in the effect of alcohol on gait in 100 participants—50 females and 50 males. Furthermore, we also investigated whether anthropometric characteristics and habitual alcohol use screened by the AUDIT questionnaire are correlated with gait parameters at 0.00% and 0.11% BrAC and whether the subjective score of drunkenness is correlated with differences in gait between sober and 0.11% BrAC conditions. We hypothesized that (i) anthropometric characteristics will show a stronger association with gait at 0.00% BrAC; (ii) no significant association between the AUDIT score and gait parameters will be observed as no participant had alcohol dependence problems; (iii) the subjective drunkenness score will show a stronger association with gait parameters in females; and (iv) greater gait impairments at 0.11% BrAC will be observed for backward gait and they will be observed in women as women have been reported to be more sensitive to alcohol intoxication.

## Methods

The study group consisted of 100 participants aged between 20 to 35 years, 50 females and 50 males. Recruitment was conducted through information about the study on social media among college students. Their characteristics are shown in Table 1. Exclusion criteria included any disease for which alcohol intake is not recommended, the taking of any medication except contraceptives at the time of data collection, pregnancy, cardiovascular disease, serious injuries of the lower limbs and balance problems, and a score on the Alcohol Disorders Identification Test (AUDIT) indicating possible alcohol dependence (a score of more than 20). Habitual alcohol use was screened using the AUDIT questionnaire developed by the Word Health Organization. AUDIT monitors hazardous use, dependence symptoms and harmful use of alcohol with a score ranging from 0 to 40^24^. Written informed consent was obtained from all participants prior to data collection. The study was approved by the local ethics committee and was performed in accordance with the Declaration of Helsinki.

**Table 1:** Participants’ characteristics.

	Females	Males	*p*
Body height (cm)	167.47 (6.21)	180.61 (6.92)	0.001
Body weight (kg)	64.45 (9.38)	82.90 (12.37)	0.001
Age (years)	24.51 (3.53)	27.08 (3.67)	0.001
BMI (kg/m^2^)	22.96 (3.05)	25.41 (3.55)	0.001
AUDIT (score)	8.12 (3.48)	10.46 (5.44)	0.026

*n*_*Female*_ = 50, *n*_Male_ = 50, descriptive data are presented as mean and standard deviation.

Participants were instructed not to consume any alcohol 24 h prior to data collection and not to consume caffeine drinks 2 h prior to data collection as caffeine increases the positive reinforcing effects of alcohol^25^. Additionally, participants were asked to eat only a light lunch on the day of data collection and not to eat 2 h before data collection.

### Procedures

**Figure 1:**
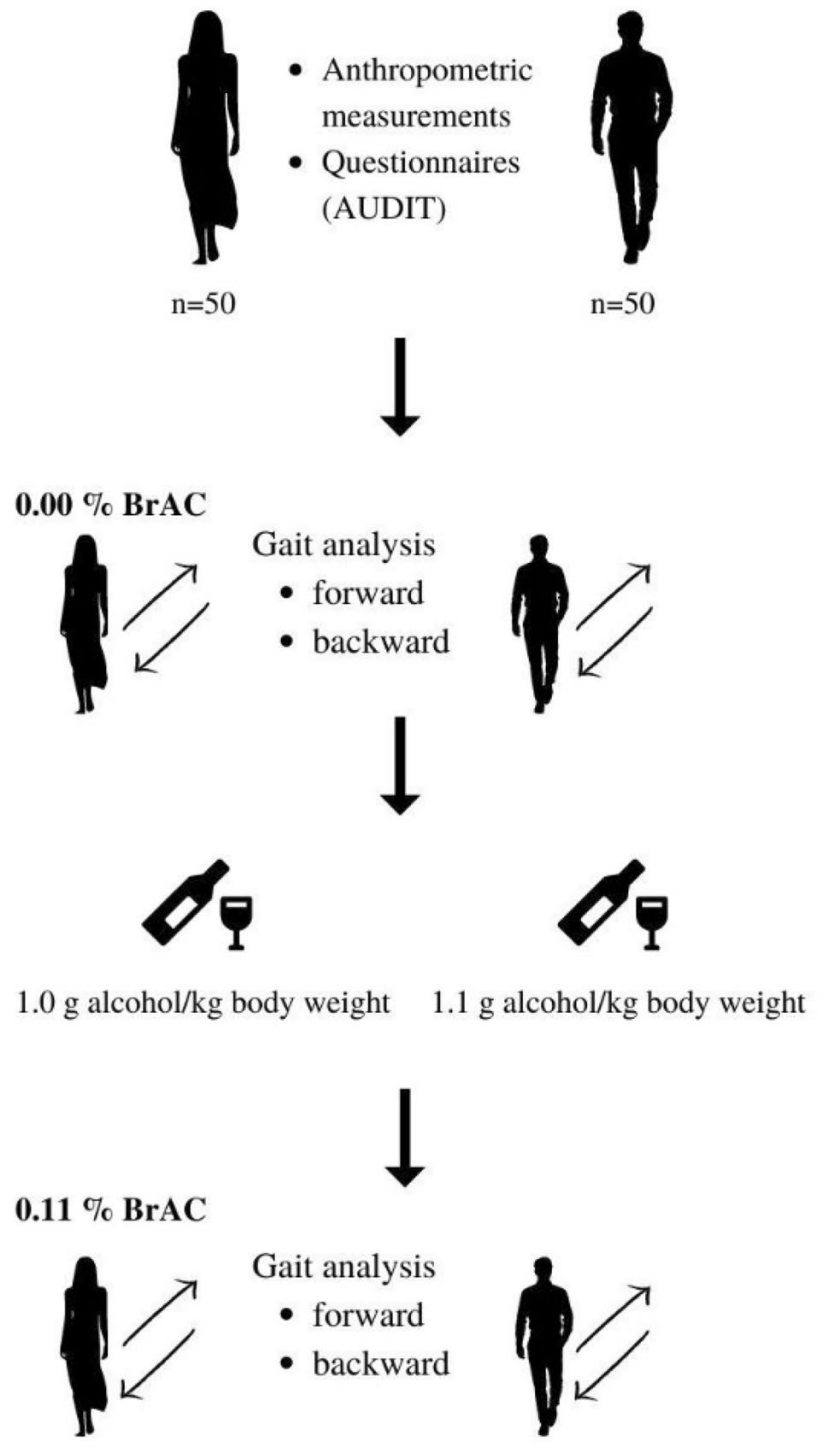
The data collection design. Canva. https://www.canva.com/.

Gait analysis was conducted twice—at the start of data collection when participants were sober and then as close as possible to a BrAC of 0.11% (i.e. 1.1 mg alcohol/L). A BrAC of 0.11% was chosen as this corresponds to stage 3 of drunkenness (described as excitement). This stage of drunkenness is characterized by a notable delay in reaction time and impaired perception and coordination^26^. Data collections were performed between 13.00 and 20.00 h from June to October 2021. A diagram of the data collection process is shown in Fig. 1.

Gait analysis was performed using a Zebris platform (FDM; GmbH, Munich, Germany). The Zebris force distribution measurement platform with built-in capacitive sensors enables the measurement and analysis of the force distribution under the feet (sensor area: 149 × 54.2 cm; number of sensors: 11,264; sampling rate: 100 Hz). Participants were asked to walk barefoot across the platform at their natural speed. The platform was placed in a custom-designed dense 10-m-long walkway surrounding the platform to provide a level walking surface. Each participant was instructed to walk forwards towards the end of the walkway, then make a 180° turn and continue walking over the platform. This cycle was repeated several times to obtain five footprints of the right foot and five footprints of the left foot. This methodology has also been used in previous studies by Kasović et al.^27^ and Štefan et al.^28^. The same instructions were used for backward gait. Participants were instructed to walk backwards toward the end of the walkway, then make a 180° turn and continue walking backward over the platform. This cycle was repeated several times as for forward gait in order to obtain five footprints of the right foot and five footprints of the left foot.

Zebris software generated data regarding gait foot rotation (°; angle between the longitudinal axis of the foot and the walking direction), stride length (cm; distance between two heel contacts of the same foot), step width (cm; distance between right and left foot), stance phase (% of the gait cycle; phase of the gait cycle when the foot is in contact with the ground), load response (% of the gait cycle; phase of the gait cycle between the initial ground contact and contralateral toe-off), single limb support (% of the gait cycle; phase of the gait cycle when only one foot is in contact with the ground), pre-swing phase (% of the gait cycle; phase of the gait cycle between the initial contact of the contralateral foot and toe-off of the other foot), swing phase (% of the gait cycle; phase of the gait cycle when the foot has no contact with the ground), double stance phase (% of the gait cycle; phase of the gait cycle when both feet are in contact with the ground), stride time (s; time between two heel contacts of the same foot), cadence (steps/min; step frequency), and velocity (km/h; average gait speed).

In addition, all participants reported their age, and their body height and weight were measured with a portable stadiometer and scale. After the first data collection, participants were asked to drink the given amount of alcohol and the second data collection was performed as close as possible to a BrAC of 0.11%. The second data collection was identical to the first data collection.

Alcohol intoxication occurred after the first (sober) data collection. The participants had 30 min in a quiet environment to drink vodka mixed with orange juice (1:1). The amount of alcohol provided to each participant was based on gender and body weight. Women received 1.0 g alcohol/kg body weight and men received 1.1 g alcohol/kg body weight. After the drink was finished, the BrAC was measured every 10 min using a Dräger Alcotest 6820 handheld breath alcohol test device (Drägerwerk AG & Co. KGaA, Germany; test range: 0 to 2.5 mg/L). This device conforms to the standard EN 15964 and has been approved for BrAC measurements by the Police of Czech Republic.

A plateau phase was identified in the BrAC recordings when no further tendency of ascending values was observed as the given amount of vodka was aimed to peak at 0.15% BrAC. In the descending phase, the second data collection of forward and backward gait was performed as close as possible to a BrAC of 0.11%. The mean BrAC in the second data collection was 0.11 ± 0.01%, and no significant differences were observed between females and males in the BrAC in the second data collection (p = 0.646). A BrAC of 0.11% (after the plateau phase was reached) was observed to occur 80 to 140 min after the initiation of drinking in our participants.

Before the second data collection, participants were asked to provide a subjective drunkenness score using a visuo-analog scale ranging from ‘sober’ to ‘extremely drunk’. Participants marked a number ranging from 1 (sober) to 100 (extremely drunk). A similar scale has also been used in a previous study to obtain a subjective score of drunkenness^29^. The subjective drunkenness score (drunkenness score), the volume of vodka provided to females and males, and the BrAC in the second data collection are shown in Table 2.

**Table 2:** Characteristics of alcohol intoxication.

	Females	Males	*p*
Drunkenness score (%)	54.20 (18.76)	46.52 (15.85)	0.024
Vodka (ml)	201.41 (29.33)	287.63 (43.96)	0.001
BrAC (ml/L)	1.09 (0.07)	1.07 (0.11)	0.646

*n*_*Female*_ = 50, *n*_Male_ = 50, descriptive data are presented as mean and standard deviation.

### Statistical analysis

The relationship between anthropometric characteristics and habitual alcohol use and gait parameters at a BrAC of 0.00% and 0.11% was tested by Spearman’s Rho (0.00–0.19: very weak, 0.20–0.39: weak, 0.40–0.59: moderate, 0.60–0.79: strong, 0.80–1.00: very strong correlation).

We performed a multivariate analysis (two-way MANOVA) for forward and backward gait to reveal potential factor interactions. The two independent variables (IV) were gender (male, female) and breath alcohol concentration (BrAC; 0.00%, 0.11%). We used the twelve gait parameters of forward and backward gait as dependent variables (DV). Due to the violation of assumptions, we used Pillai’s trace results for multivariate tests.

Most of the variables did not meet the assumptions of normal distribution tested by the Shapiro–Wilk test. The Mann–Whitney U test was used to compare the differences between genders when walking forward and backward during the data collections at a BrAC of 0.00% and 0.11% and to compare the characteristics of the participants. The Wilcoxon Signed Ranks test was used to compare the differences between sober (0.00% BrAC) and intoxicated (0.11% BrAC) conditions. Bonferroni correction (α_Bon_ = 0.0042) was applied to an overall *α* level of 0.05 to avoid Type I error.

To reduce the risk of the first type of error, we further calculated the effect size index r, which reflects the empirical basis of the effects of the real population^30,31^. The magnitudes of the effect size index r can be interpreted as a small effect (r = 0.1), a medium effect (r = 0.3) or a large effect (r = 0.5)^30^. SPSS Statistics (IBM Corp. Released 2021. IBM SPSS Statistics for Windows, Version 28.0. Armonk, NY: IBM Corp) was used for the statistical analysis.

The relationship between the subjective drunkenness score and differences in gait parameters recorded at a BrAC of 0.00% and a BrAC of 0.11% was tested by Spearman’s Rho.

### Ethics approval and consent to participate

This study was carried out according to the Declaration of Helsinki guidelines and was approved by the Masaryk University Research Ethics Committee (EKV-2021-007). Informed consent was obtained from all subjects involved in the study.

## Results

### Anthropometrical characteristics

Body weight and body height differed significantly between women and men (p = 0.001). A moderate correlation was observed between anthropological characteristics and gait parameters only in forward gait when sober. In women, body height was moderately correlated with stride length (rho = 0.429, p = 0.002; CI 0.163, 0.637). In males, body weight was moderately correlated with stride time (rho = 0.410, p = 0.003, CI 0.140, 0.623) and cadence (rho = − 0.416, p = 0.003, CI − 0.628, − 0.148).

### AUDIT score

The AUDIT score differed significantly between women and men (p = 0.026). No moderate or strong correlation was observed between the AUDIT score and gait parameters at a BrAC of 0.00% and a BrAC of 0.11%. In women, a weak correlation was observed in forward gait when walking at 0.00% BrAC between the AUDIT score and the stride time (rho = 0.281, p = 0.048, CI − 0.006, 0.525) and cadence (rho = − 0.289, p = 0.041, CI − 0.532, − 0.004) and at 0.11% BrAC between the AUDIT score and the stride time (rho = 0.292, p = 0.040, CI 0.006, 0.534) and cadence (rho = − 0.283, p = 0.046, CI − 0.527, 0.003). No statistically significant correlation was observed in men. No correlation was observed between the AUDIT score and the backward gait parameters.

### Multivariate analysis

The results of two-way MANOVA are shown in Table 3. For forward gait, MANOVA showed a statistically significant difference in gender (F (11, 185) = 12.70, Pillai’s Trace = 0.43, partial η^2^ = 0.430) indicating that 43.0% of the variance in gait parameters can be explained by gender. No statistically significant differences were observed in the case of BrAC and the combination of gender and BrAC.

**Table 3:** Multivariate tests for forward gait.

Effect	F	p	Partial η^2^	Observed power
Gender	12.70	< 0.001	0.430	1.000
BrAC	1.11	0.355	0.062	0.601
Gender × BrAC	0.68	0.758	0.039	0.367

Levene’s test of the equality of error variances showed that the homogeneity of variance cannot be assumed for most dependent variables. Therefore, an alpha level of 0.001 was used when evaluating univariate ANOVAs as recommended by Allen and Bennett^32^. The results of the univariate test are shown in Supplementary material (S1). Step width, foot rotation, cadence and stride time explain 18.2%, 17.4%, 9.5% and 7.9% of variance in dependent variables, respectively.

For backward gait, MANOVA showed a statistically significant difference in gender (F (11,186) = 5.70, Pillai’s trace = 0.25, partial η^2^ = 0.251) indicating that 25.1% of the variance in gait parameters can be explained by gender. No statistically significant differences were observed in the case of BrAC and the combination of gender and BrAC (Table 4). The results of the univariate test for backward gait are shown in Supplementary material (S2). Foot rotation and step width explain 8.2% and 7.2% of the variance in the dependent variables, respectively.

**Table 4:** Multivariate tests for backward gait.

Effect	F	p	Partial η^2^	Observed power
Gender	5.70	< 0.001	0.25	1.000
BrAC	1.09	0.376	0.06	0.588
Gender × BrAC	0.62	0.808	0.04	0.336

### Two sample tests

#### Forward gait

The medians, minimums and maximums for women and men during forward gait when walking sober and after alcohol intoxication are shown in Table 5. The results of the statistical analysis for forward gait are shown in Supplementary material (S3).

**Table 5:** Forward gait at a BrAC of 0.00% and 0.11% in females and males.

	0.00% BrAC		0.11% BrAC	
	Females	Males	Females	Males
Foot rotation. °	3.24 (4.06)	7.87 (5.84)	3.75 (4.0)	7.8 (4.97)
Stride length. cm	128.40 (12.40)	135.07 (13.42)	134.96 (13.97)	138.47 (14.75)
Step width. cm	9.26 (2.16)	12.09 (2.8)	9.57 (2.49)	11.96 (3.56)
Stance phase. %	63.04 (2.12)	62.55 (2.19)	61.95 (2.36)	62.34 (2.55)
Load response. %	12.77 (1.78)	12.8 (2.13)	12.42 (2.02)	12.32 (2.23)
Single limb support. %	37.39 (2.17)	37.49 (2.34)	37.49 (1.95)	37.67 (2.58)
Pre-swing. %	12.89 (1.71)	12.24 (1.88)	12.09 (1.72)	12.35 (2.27)
Swing phase. %	36.96 (2.12)	37.45 (2.19)	38.05 (2.36)	37.66 (2.55)
Double stance phase. %	25.66 (3.06)	25.03 (3.55)	24.51 (3.28)	24.68 (4.17)
Stride time. sec	1.12 (0.10)	1.18 (0.09)	1.13 (0.10)	1.19 (0.09)
Cadence. steps/min	107.58 (9.45)	102.20 (7.53)	106.78 (8.88)	101.55 (6.96)
Velocity. km/h	4.17 (0.65)	4.14 (0.48)	4.33 (0.68)	4.22 (0.49)

Descriptive data are presented as mean and standard deviation.

#### Gender differences

When walking forward, both sober (0.00% BrAC) and intoxicated (0.11% BrAC), statistically significant gender differences were observed after Bonferroni correction. A smaller foot rotation (p =  < 0.001 at 0.00% BrAC, p =  < 0.001 at 0.11% BrAC), shorter step width (p =  < 0.001 at 0.00% BrAC, p =  < 0.001 at 0.11% BrAC), shorter stride length (p = 0.002 at 0.00% BrAC, p =  < 0.001 at 0.11% BrAC) and higher cadence (p = 0.002 at 0.00% BrAC, p =  < 0.001 at 0.11% BrAC) were observed in females as compared to males. Females seem to compensate for shorter step length with higher cadence to achieve the same self-selected gait velocity (4.17 ± 0.65) observed in males (4.14 ± 0.48).

#### Females

No statistically significant differences were observed when the 0.00% BrAC and 0.11% BrAC forward gait were compared in females.

#### Males

Statistically significant differences were observed in foot rotation (p = 0.001), which decreased during intoxication with alcohol, when the 0.00% BrAC and 0.11% BrAC forward gait were compared in males.

#### Backward gait

The medians, minimums and maximums and the results of the statistical analysis for women and men during backward gait when walking sober and after alcohol intoxication are shown in Table 6. The results of the statistical analysis for backward gait are shown in Supplementary material (S4).

**Table 6:** Backward gait at 0.00% and 0.11% BrAC in females and males.

	0.00% BrAC		0.11% BrAC	
	Females	Males	Females	Males
Foot rotation. °	− 1.89 (11.50)	− 8.29 (6.57)	− 3.37 (6.03)	− 7.05 (8.78)
Stride length. cm	99.15 (10.38)	104.59 (12.87)	104.46 (11.36)	104.83 (15.46)
Step width. cm	12.55 (3.17)	14.94 (3.53)	13.89 (3.69)	15.68 (4.59)
Stance phase. %	64.02 (3.23)	64.48 (2.21)	64.09 (3.90)	65.25 (2.80)
Load response. %	14.23 (1.57)	14.63 (1.80)	13.84 (2.47)	14.91 (2.60)
Single limb support. %	35.53 (1.91)	35.21 (2.30)	36.08 (4.66)	35.09 (4.38)
Pre-swing. %	14.26 (3.04)	14.73 (1.86)	14.22 (2.04)	15.14 (2.04)
Swing phase. %	35.98 (3.23)	35.52 (2.21)	35.91 (3.90)	34.75 (2.80)
Double stance phase. %	28.49 (3.72)	29.18 (3.17)	27.99 (3.80)	29.89 (4.27)
Stride time. sec	1.16 (0.13)	1.19 (0.11)	1.16 (0.15)	1.21 (0.14)
Cadence. steps/min	104.91 (11.82)	102.08 (8.99)	105.07 (12.93)	100.32 (10.86)
Velocity. km/h	3.13 (0.61)	3.20 (0.53)	3.29 (0.64)	3.16 (0.59)

Descriptive data are presented as mean and standard deviation.

#### Gender differences

Statistically significant gender differences were observed after Bonferroni correction when walking backward in a sober condition (0.00% BrAC). Smaller foot rotation (p =  < 0.001) and narrower step width (p =  < 0.001) were observed in females as compared to males. A higher foot rotation was observed (p = 0.002) in males as compared to females when walking at 0.11% BrAC.

#### Females

When 0.00% BrAC and 0.11% BrAC backward gait was compared in women, statistically significant differences were observed in stride length (p =  < 0.001), which increased, double stance phase (p = 0.002), which decreased, and velocity (p =  < 0.001), which increased, during the 0.11% BrAC data collection.

#### Males

No statistically significant differences were observed in males when comparing backward gait at 0.00% BrAC and 0.11% BrAC.

The summary of the effect size index r for differences between 0.00% and 0.11% BrAC in forward and backward gait in females and males is shown in Supplementary material (S5). The magnitude of the effect size index r fell within the range of a trivial (r = − 0.01) to large (r = − 0.52) effect. A medium and large effect reflected statistical significance in the variables analyzed.

### Subjective score of drunkenness

The mean subjective score of drunkenness for females was 54.20 ± 18.91 (ranging from 5 to 90), while that for males was 46.52 ± 15.97 (ranging from 5 to 80). The subjective drunkenness score differed significantly between women and men (p = 0.024).

The subjective score of drunkenness was correlated with the differences (dif.) for each gait parameter between the 0.00% and 0.11% BrAC data collection; only weak correlations were, however, observed.

When walking forward, the subjective score of drunkenness showed a statistically significant correlation with foot rotation dif. (rho = 0.317, p = 0.025) in females. No correlation was observed between the subjective score of drunkenness and forward gait parameters in males.

When walking backwards, the subjective score of drunkenness showed statistically significant correlation with stride length dif. (rho = − 0.345, p = 0.014), stance phase dif. (rho = 0.297, p = 0.036), pre-swing dif. (rho = 0.377, p = 0.007), swing phase dif. (rho = − 0.296, p = 0.037), double stance phase dif. (rho = 0.346, p = 0.014) and velocity dif. (rho = − 0.389, p = 0.005) in females. The subjective score of drunkenness showed statistically significant correlation with load response dif. (rho = 0.332, p = 0.018) in males when walking backward.

**Figure 2:**
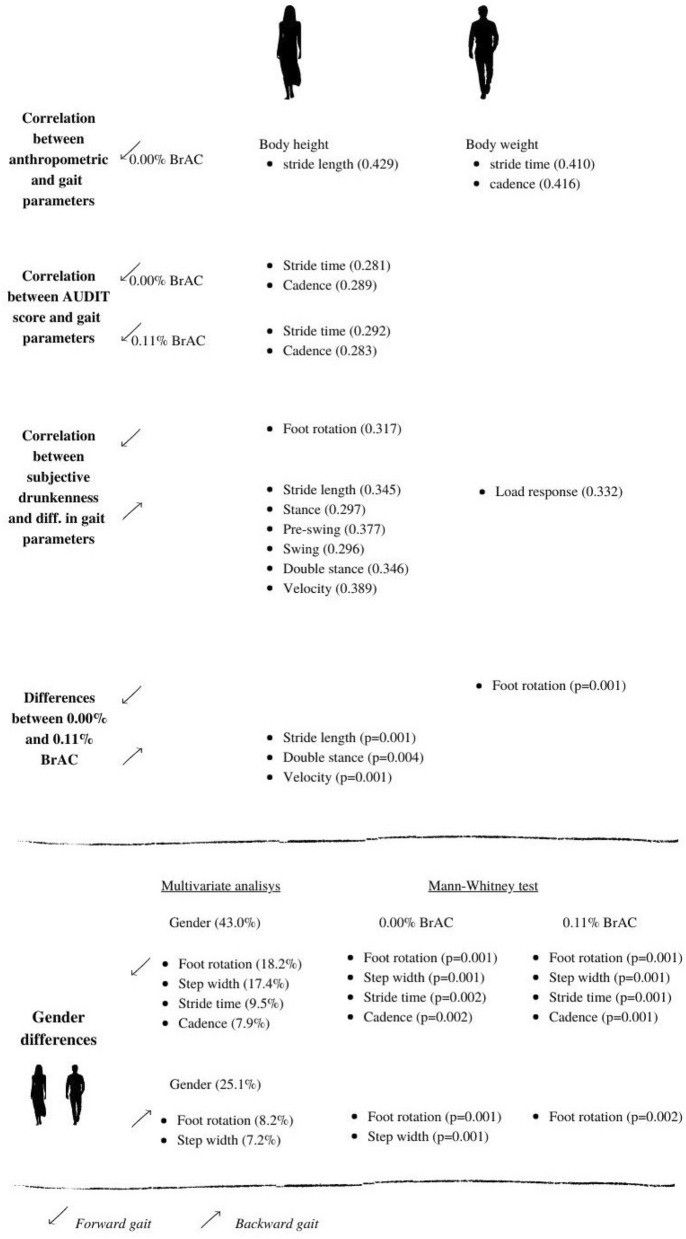
Summary of the results. Canva. https://www.canva.com/.

The main results are summarized in Fig. 2. Figure 2 shows correlations between gait parameters and anthropometric characteristics, the AUDIT score and subjective drunkenness, the main differences being found in gait at 0.00% and 0.11% BrAC and gender differences.

## Discussion

Our study analyzed the gender differences in forward and backward gait when sober and at a BrAC of 0.11% in one hundred participants—fifty females and fifty males. Our study also investigated whether anthropometric characteristics and the AUDIT score correlate with gait at 0.00% and 0.11% BrAC and whether the subjective score of drunkenness correlates with differences in gait parameters between a BrAC of 0.00% and 0.11%. The results of our study show that alcohol intoxication has a greater impact on gait in females who tended to show an increase in balance-related variables with an increase in the subjective score of drunkenness. Females have previously been observed to be more sensitive to the behavioral effects of ethanol^33^, as cortical gray matter perfusion is affected differently in females and males, probably due to differences in the hormonal, metabolic and hemodynamic autoregulatory systems^34^. No previous study focusing on gender differences in the effect of alcohol intoxication on gait is known to the authors.

### Anthropometric characteristics

Anthropometric characteristics have been described as affecting temporospatial gait parameters and postural control^35–38^. In most previous studies, stride length and gait velocity have been associated with body height and body weight, respectively^35,36^. However, some of the previous studies show different effects of anthropometric parameters on gait in females and males (e.g.^37^), as anatomical dimensions differ between the genders^39^. The results of our study confirm the correlation between body height and stride length in females and the correlation between body weight and stride time and cadence in males when sober. However, no significant correlation was observed between anthropometric characteristics and gait after alcohol intoxication in this study, suggesting a greater effect of alcohol intoxication as compared to anthropometric characteristics on gait impairments at a BrAC of 0.11%.

### AUDIT score

The AUDIT score monitors the hazardous use, dependence symptoms and harmful use of alcohol^24^. Hazardous alcohol consumption is the most prevalent public health problem in young adults^40^ as young adults have been reported to have the highest prevalence of alcohol disorders and hazardous alcohol consumption^40–43^. Alcohol use disorder has been found to affect postural stability and gait^44,45^ as ethanol-induced cerebellar atrophy is strongly associated with impaired gait and balance^46^. Healthy participants without alcohol use disorder were enrolled in our study. A weak correlation was observed between the AUDIT score and stride time and cadence at a BrAC of 0.00% and 0.11% in women, suggesting that women may be more sensitive to the adverse effects of habitual alcohol use. In young adults, higher alcohol consumption and hazardous drinking has been observed in males as compared to females^47^. In accordance with this, a higher AUDIT score was observed in male participants as compared to female participants in our study.

### Differences between a BrAC of 0.00% and 0.11%

Foot placement is the dominant mechanism of stability control during gait^48^ and an increase in step width and foot rotation leads to the maintenance of stability in challenging conditions^49^. An increase in foot rotation was observed for a BrAC of 0.11% in men when walking forward to maintain the stability of their gait. In our study, no similar mechanism for increasing stability when intoxicated with alcohol was observed in women. The amount of alcohol consumed in our study appears not to be sufficient to induce cerebellar gait ataxia in forward gait. Gait can be generated volitionally or automatically. Subcortical structures such as the brainstem and basal ganglia play a role in automatic walking, and the cerebral cortex is involved with a higher level of effort^50^. The backward gait constitutes a greater challenge to the nervous system compared to the forward gait. It has been assumed that forward and backward gait have the same neural mechanism, although backward gait control networks tend to be more affected by aging, intellectual disability, Parkinson’s disease and dual cognitive tasks, suggesting independent control systems for forward and backward gait^13,51–55^. The risk of falling increases with alcohol intoxication^56^. Additionally, many falls occur when stepping backward^52^. Backward gait analysis was evaluated in our study as impairments to backward stepping may increase the risk of falls and related injuries when intoxicated with alcohol.

When backward gait was compared in females at a BrAC of 0.00% and 0.11%, an increase in stride length, a prolonged single-limb support phase, a shorter double-stance phase and an increase in velocity were observed during the 0.11% BrAC data collection. The decrease in the duration of the stance phase and the increase in stride length were associated in a previous study with an increase in gait velocity^57^. The increase in gait velocity produces a more unstable backward gait. Research in movement decision-making postulates that people generate movement at higher velocities to acquire a greater amount of reward. Motor commands guide the balance of movement between stable gait, when moving slowly with better precision, and the desire to maximize the reward, i.e. to move quickly and get reward sooner^58,59^. When intoxicated with alcohol, females may try harder to acquire the reward than when sober, i.e. to complete the given task. No statistically significant differences between a BrAC of 0.00% and 0.11% were observed in males.

### Gender differences

Multivariate analysis showed a statistically significant difference in gender indicating that 43.0% of the variance in forward gait and 25.1% of the variance in backward gait can be explained by gender. In forward gait, the foot rotation, step width, stride time and cadence explain most of the variance in gait parameters. In backward gait, most of the variance in gait parameters can be explained by foot rotation and step width. The results of the Mann–Whitney test show that when comparing forward gait in females and males, females performed significantly smaller foot rotation, smaller step width, shorter stride time and higher cadence compared to males at a BrAC of both 0.00% and 0.11%. Women seem to compensate for shorter step length with a higher cadence to achieve the same natural gait velocity as observed in men, as has also been observed in a previous study comparing gait in women and men^20^. Shorter stride length and narrower step width have also been observed in women as compared to men in forward gait in previous studies^18,19^. However, differences in stride length do not seem to be statistically significant when normalized by body height^20^.

When walking backward, a slower gait velocity and a shorter stride length were observed in women as compared to men^19^. In our study, women performed significantly smaller foot rotation (at a BrAC of 0.00% and 0.11%) and narrower step width (at a BrAC of 0.00%) as compared to men, i.e. differences related to a decrease in gait stability.

### Subjective score of drunkenness

The subjective score of drunkenness differed significantly between women and men. Higher levels of subjective intoxication have previously been observed in females as compared to males^33,37^. However, only a weak correlation was observed between the subjective drunkenness score and gait parameters, primarily in women who tended to increase balance-related gait variables when subjectively feeling more drunk. Previous studies show that the expectation of impairment caused by alcohol intoxication contributes to these impairments. Gender differences in expectancies of alcohol effects have been observed in many aspects of human behavior^60,61^ and may lead to a greater impact of alcohol intoxication on gait in women and consequently to a stronger correlation between the subjective score of drunkenness and changes in gait parameters as compared to men.

### Limitations and future work

There are several limitations to our study. A statistically significant difference in age between female and male participants was observed despite the focus of our study on young adults aged 20 to 35 years. Furthermore, our study included only healthy young participants, for which reason different gait impairments caused by alcohol intoxication may be observed in older adults and the elderly. The order of 0.00% and 0.11% BrAC gait data collections may be considered a confound because the backward gait is a novel task and there may have been some learning effects during the 0.00% BrAC data collection. Also, all data collections were performed during barefoot walking which may not be representative of the typical environment of daily life when young adults drink alcohol, especially in young women who are used to wearing high heels or other fashionable shoes when going out for a drink. Given that the results of our study showed gender differences in gait when intoxicated with alcohol, future research focused on women and the effect of footwear, history of falls and alcohol intoxication on gait parameters that may affect the risk of falls would provide more detailed knowledge about this topic.

## Conclusions

To conclude, the results of our study show that gender differences in gait persist after alcohol intoxication in young adults. Different strategies to maintain stability during gait were observed in women and men. A significant decrease in stability at a blood alcohol concentration of 0.10% has been observed in a previous study^29^. Males increase their foot rotation in the forward gait when intoxicated with alcohol. A decrease in balance-related variables was observed in females when walking backward at a BrAC of 0.11%, but no statistically significant change was observed in males. Additionally, females tended to perform an increase in balance-related gait variables in both forward and backward gait when subjectively feeling more drunk.

## Supplementary Information

Supplementary Information.

## Data Availability

The datasets used and/or analyzed during the current study are available from the corresponding author on reasonable request.
