# Supplementary material for: Gender differences in the effect of a 0.11% breath alcohol concentration on forward and backward gait
Source: Sci Rep. 2022 Nov 5;12:18773. doi: 10.1038/s41598-022-23621-y (PMC9637089; doi:10.1038/s41598-022-23621-y)
Supplement: Supplementary file 1 — Supplementary Information. [file 41598_2022_23621_MOESM1_ESM.pdf]

S1. Univariate test results for forward gait (IV=gender).

DV	Sum of Squares	Mean Square	F	p	Partial η^2	Observed Power
Foot rotation, °	937.228	937.228	41.056	<.001	0.174	1.000
Stride length, cm	1289.299	1289.299	6.907	0.009	0.034	0.744
Step width, cm	339.813	339.813	43.282	<.001	0.182	1.000
Stance phase, %	0.135	0.135	0.025	0.874	0.000	0.053
Load response, %	0.078	0.078	0.019	0.892	0.000	0.052
Single limb support, %	0.929	0.929	0.18	0.672	0.001	0.071
Pre-Swing, %	1.963	1.963	0.539	0.464	0.003	0.113
Swing phase, %	0.135	0.135	0.025	0.874	0.000	0.053
Double stance phase, %	2.735	2.735	0.218	0.641	0.001	0.075
Stride time, s	0.154	0.154	16.823	<.001	0.079	0.983
Cadence, steps/min	1399.932	1399.932	20.502	<.001	0.095	0.995
Velocity, km/h	0.254	0.254	0.754	0.386	0.004	0.139

S2. Univariate test results for backward gait (IV=gender).

DV	Sum of Squares	Mean Square	F	p	Partial η^2	Observed Power
Foot rotation, °	1268.427	1268.427	17.562	<.001	0.082	0.986
Stride length, cm	422.355	422.355	2.633	0.106	0.013	0.365
Step width, cm	218.328	218.328	15.274	<.001	0.072	0.973
Stance phase, %	32.882	32.882	3.427	0.066	0.017	0.453
Load response, %	27.285	27.285	5.889	0.016	0.029	0.675
Single limb support, %	21.377	21.377	1.714	0.192	0.009	0.256
Pre-Swing, %	24.354	24.354	4.637	0.033	0.023	0.573
Swing phase, %	32.882	32.882	3.427	0.066	0.017	0.453
Double stance phase, %	84.340	84.340	5.968	0.015	0.030	0.681
Stride time, s	0.074	0.074	4.235	0.041	0.021	0.535
Cadence, steps/min	718.319	718.319	5.680	0.018	0.028	0.660
Velocity, km/h	0.044	0.044	0.126	0.723	0.001	0.064

S3. Forward gait at 0.00% and 0.11% BrAC conditions in females and males.

	**p (gender dif. at 0.00% BrAC)**	**p (gender dif. at 0.11% BrAC)**	**p (females, comparison between 0.00 and 0.11% BrAC)**	**p (males, comparison between 0.00 and 0.11% BrAC)**
Foot rotation. °	<0.001*	<0.001*	0.484	0.001*
Stride length. cm	0.006	0.167	0.572	0.401
Step width. cm	<0.001*	<0.001*	0.025	0.508
Stance phase. %	0.132	0.620	0.013	0.308
Load response. %	0.615	0.482	0.806	0.496
Single limb support. %	0.807	0.566	0.498	0.140
Pre-Swing. %	0.069	0.684	0.295	0.020
Swing phase. %	0.132	0.620	0.013	0.308
Double stance phase. %	0.201	0.863	0.308	0.088
Stride time. sec	0.002*	<0.001*	0.057	0.134
Cadence. steps/min	0.002*	<0.001*	0.052	0.249
Velocity. km/h	0.885	0.391	0.521	0.197

** Highlights statistical significance after Bonferroni correction*

S4. Backward gait at 0.00% and 0.11% BrAC conditions in females and males.

	**p (gender dif. at 0.00% BrAC)**	**p (gender dif. at 0.11% BrAC)**	**p (females, comparison between 0.00 and 0.11% BrAC)**	**p (males, comparison between 0.00 and 0.11% BrAC)**
Foot rotation. °	<0.001*	0.002*	0.215	0.043
Stride length. cm	0.015	0.738	<0.001*	0.031
Step width. cm	<0.001*	0.070	0.357	0.896
Stance phase. %	0.172	0.011	0.211	0.675
Load response. %	0.329	0.111	0.032	0.253
Single limb support. %	0.620	0.588	0.048	0.069
Pre-Swing. %	0.229	0.030	0.027	0.873
Swing phase. %	0.172	0.011	0.211	0.675
Double stance phase. %	0.393	0.039	0.004*	0.633
Stride time. sec	0.258	0.057	0.694	0.339
Cadence. steps/min	0.313	0.036	0.599	0.352
Velocity. km/h	0.275	0.434	<0.001*	0.131

** Highlights statistical significance after Bonferroni correction*

S5. Summary of the Effect size index r and statistical significance.

	**Forward gait**				**Backward gait**			
	**Female x Male**		**0.00% x 0.11% BrAC**		**Female x Male**		**0.00% x 0.11% BrAC**	
	**0.00% BrAC**	**0.11% BrAC**	**Female**	**Male**	**0.00% BrAC**	**0.11% BrAC**	**Female**	**Male**
Foot rotation. °	-0.46**	-0.39**	-0.18*	-0.33**	-0.35**	-0.31**	-0.12*	-0.20*
Stride length. cm	-0.28*	-0.14*	-0.06	-0.08	-0.24*	-0.03	-0.52***	-0.22*
Step width. cm	-0.49**	-0.36**	-0.22*	-0.07	-0.34**	-0.18*	-0.09	-0.01
Stance phase. %	-0.15*	-0.05	-0.25*	-0.10*	-0.14*	-0.25*	-0.13*	-0.04
Load response. %	-0.05	-0.07	-0.02	-0.07	-0.10*	-0.16*	-0.21*	-0.11*
Single limb support. %	-0.02	-0.06	-0.07	-0.15*	-0.05	-0.05	-0.20*	-0.18*
Pre-Swing. %	-0.18*	-0.04	-0.10*	-0.23*	-0.12*	-0.22*	-0.22*	-0.02
Swing phase. %	-0.15*	-0.05	-0.25*	-0.10*	-0.14*	-0.25*	-0.13*	-0.04
Double stance phase. %	-0.13*	-0.02	-0.10*	-0.17*	-0.09	-0.21*	-0.29*	-0.05
Stride time. sec	-0.32**	-0.36**	-0.19*	-0.15*	-0.11*	-0.19*	-0.04	-0.10*
Cadence. steps/min	-0.32**	-0.36**	-0.19*	-0.12*	-0.10*	-0.21*	-0.05	-0.09
Velocity. km/h	-0.01	-0.09	-0.06	-0.13*	-0.11*	-0.08	-0.41**	-0.15*

** Small Effect size, ** medium effect size, ***large effect size*
